# A novel method for predicting cell abundance based on single-cell RNA-seq data

**DOI:** 10.1186/s12859-021-04187-4

**Published:** 2021-08-25

**Authors:** Jiajie Peng, Lu Han, Xuequn Shang

**Affiliations:** School of Computer Science, Northwestern Polytechnical University, Chang’an Ave, Changan Qu, Xi’an City, Shaanxi Province China

**Keywords:** Deconvolution, Bioinformatics, Cell abundance prediction, Weighted least squares

## Abstract

**Background:**

It is important to understand the composition of cell type and its proportion in intact tissues, as changes in certain cell types are the underlying cause of disease in humans. Although compositions of cell type and ratios can be obtained by single-cell sequencing, single-cell sequencing is currently expensive and cannot be applied in clinical studies involving a large number of subjects. Therefore, it is useful to apply the bulk RNA-Seq dataset and the single-cell RNA dataset to deconvolute and obtain the cell type composition in the tissue.

**Results:**

By analyzing the existing cell population prediction methods, we found that most of the existing methods need the cell-type-specific gene expression profile as the input of the signature matrix. However, in real applications, it is not always possible to find an available signature matrix. To solve this problem, we proposed a novel method, named DCap, to predict cell abundance. DCap is a deconvolution method based on non-negative least squares. DCap considers the weight resulting from measurement noise of bulk RNA-seq and calculation error of single-cell RNA-seq data, during the calculation process of non-negative least squares and performs the weighted iterative calculation based on least squares. By weighting the bulk tissue gene expression matrix and single-cell gene expression matrix, DCap minimizes the measurement error of bulk RNA-Seq and also reduces errors resulting from differences in the number of expressed genes in the same type of cells in different samples. Evaluation test shows that DCap performs better in cell type abundance prediction than existing methods.

**Conclusion:**

DCap solves the deconvolution problem using weighted non-negative least squares to predict cell type abundance in tissues. DCap has better prediction results and does not need to prepare a signature matrix that gives the cell-type-specific gene expression profile in advance. By using DCap, we can better study the changes in cell proportion in diseased tissues and provide more information on the follow-up treatment of diseases.

## Background

Biological tissues are often complex and consist of many morphologically similar cells and intercellular substances. For example, blood contains various cell types such as granulocyte, erythroid, megakaryocytic, and mononuclear cells [1]. It is important to understand the composition of cell types and their proportion in intact tissues, as changes in certain cell types in tissues might be the underlying causes of diseases in humans [2]. If we can describe the difference in the composition of cell type for different diseases or different subjects, we can understand the mechanism of the disease better and research the cell targets to treat the disease better [3, 4]. Based on the single-cell RNA sequencing data, the composition of cell types and their proportion in intact tissues can be estimated. With the bulk RNA-seq data of a certain type of tissue and the corresponding composition of cell types, the composition of cell types for the tissue can be predicted by the deconvolution method.

Bulk RNA-seq is a widely used method in cell sequencing. It extracts DNA from all cells in the tissue and then breaks it down into fragments [5]. The data obtained by bulk RNA-seq represents the average expression of genes across all cells in the tissue. Compared with bulk RNA-seq, single-cell sequencing uses single-cell separation technology to separate individual cells and uses optimized next-generation DNA sequencing technology (NGS) to detect the sequence of single cells and obtain gene expression profiles of individual cells [6]. Single-cell sequencing technology can obtain differences between cells in specific micro-environments to facilitate the study of their functional differences. It helps us to study different cell types, which is of great benefit to the study of developmental biology. Although single-cell sequencing can obtain the composition and abundance of cell type, it is expensive to be applied in clinical studies involving numerous subjects. Therefore, it is urgent to develop a method to infer the proportion of each cell type in the tissue, based on known cell type-specific gene expression profiles obtained from scRNA-seq data.

According to the implementation of the deconvolution method, existing methods can be broadly divided into two categories: non-negative least squares-based methods and Support Vector Regression (SVR)-based methods.

The least squares-based method is a mathematical optimization method. It finds the best function match for the data by minimizing the sum of the squares of the errors. The least-squares method can be used to obtain unknown data and minimize the sum of the squares of the errors between the obtained data and the actual data [7]. There are several deconvolution methods based on non-negative least squares, such as DeconRNASeq, MuSiC. DeconRNASeq [8] is an R package for deconvolution of heterogeneous tissues based on mRNA-seq data. It uses a globally optimized non-negative decomposition algorithm to estimate the mixing ratio of different cell types in next-generation sequencing data through quadratic programming. The input of DeconRNASeq is a cell-type-specific gene expression matrix and a mixture gene expression matrix, and the output is a cell proportion matrix. MuSiC [9] is an R package that utilizes cell-type-specific gene expression from single-cell RNA sequencing data to characterize cell type compositions from bulk RNA-seq data in complex tissues. It uses weighted non-negative least squares (W-NNLS) to implement deconvolution. The input of MuSiC is a single-cell RNA-seq dataset and a tissue gene expression matrix obtained by bulk RNA-seq, and the output is a cell occupancy matrix. MuSiC weights the non-negative least squares input matrix based on the variance of the expression of the same type of cells in different samples.

Support vector machine (SVM) is a supervised learning method used for classification and regression [10]. There are several deconvolution methods based on SVR, such as CIBERSORT, Bseq-SC, and CPM. CIBERDORT [11] is a web-based tool that uses gene expression data to estimates cell type abundance in mixed cell populations. CIBERDORT provides a signature gene file named LM22, which contains 22 different types of immune cells. If the bulk data only includes these cell types, users can use the LM22 directly and obtain the deconvolution result. If other cell types are involved in the input, users need to upload the signature gene file. Bseq-SC [12] is an R package that obtains cell type ratios based on the CIBERDORT deconvolution step and integrates the obtained ratio into cell type-specific differential analysis. CPM [13] is an R package that identifies cell abundance from a large number of gene expression data of heterogeneous samples using deconvolution based on cell population mapping. To improve the performance in the presence of a large number of reference profiles, CPM uses a consensus approach. It repeats the deconvolution method N times in N different subsets of the reference profile. The final predicted abundance result is the average of N calculation results.

There are also some cell abundance prediction methods that do not use deconvolution for prediction, such as UNDO and TIMER. UNDO [14] is an R-package for unsupervised deconvolution of mixed expression matrices of tumor stromal cells. It automatically detects cell-specific marker genes located on the scatter radius of mixed gene expression, estimates the proportion of cells in each sample, and deconvolutes the mixed expression into cell-specific expression profiles. It does not require a signature matrix that provides the cell-type-specific gene expression profile. TIMER [15] is a web-based tool for systematically assessing the clinical impact of different immune cells in specific cancers. It can estimate the abundance of six types of immune cells in the tumor microenvironment through a new statistical method.

The major limitation of existing methods is that users need to provide the signature matrix of cell-type-specific gene expression profiles. However, the signature matrix is not always available. Among the aforementioned methods, MuSiC only needs single-cell data to generate a signature matrix. Therefore, we improved the process of calculating the signature matrix and proposed a better method DCap (Deconvolution Cell abundance prediction).

## Result

### Experimental dataset

We used three datasets as experimental datasets, including two single-cell RNA sequencing datasets and one bulk RNA-seq dataset. Details are shown in Table 1.

**Table 1:** Experimental dataset

Dataset name	Dataset sources	Dataset type	Organization type	Sample size
E-MTAB-5061	Segerstolpe et al.	Single-cell RNA sequencing	Human pancreas	10 (6 normal + 4 T2D)
GSE50244	Fadista et al.	Bulk RNA-seq	Human pancreas	89
GSE81608	Xin et al.	Single-cell RNA sequencing	Human pancreas	18 (12 normal + 6 T2D)

### Evaluation metrics

Three metrics are used for evaluation: root-mean-square deviation (RMSD), mean absolute difference (mAD) and pearson product moment correlation coefficient (R).

#### Root-mean-square deviation

The root-mean-square is a measurement method used to estimate the difference between values. *RMSD* is applied to evaluate the error in the prediction. The smaller *RMSD* indicates that the predicted value is closer to the ground truth.

The calculation equation of *RMSD* is:

1 $$\begin{aligned} RMSD\left( {\hat{\alpha } } \right) = \sqrt{E\left( {{{\left( {\hat{\alpha } - \alpha } \right) }^2}} \right) } \end{aligned}$$

where $\alpha$ represents the true value and $\hat{\alpha }$ represents the predicted value.

#### Mean absolute difference

The mean absolute difference represents the average difference between the predicted value and ground truth. It is also used to express the quality of the predicted results. The smaller *mAD* represents the closer the predicted value to ground truth.

*mAD* is calculated as:

2 $$\begin{aligned} mAD\left( {\hat{\alpha } } \right) = E\left( {\left| {\hat{\alpha } - \alpha } \right| } \right) \end{aligned}$$

where $\alpha$ represents the true value and $\hat{\alpha }$ represents the predicted value.

#### Pearson correlation coefficient

Pearson product-moment correlation coefficient is applied to measure the degree of linear correlation between two variables, whose value is between $-1$ and 1. A higher correlation between the predicted value and ground truth represents the better prediction result. The higher the Pearson product-moment correlation coefficient represents better prediction results.

Pearson correlation coefficient between two variables is the quotient of variance and standard deviation between two variables. The calculation equation of *R* is:

3 $$R\left( {\hat{\alpha } ,\alpha } \right)= \frac{{cov\left( {\hat{\alpha } ,\alpha } \right) }}{{\sqrt{Var\left[ {\hat{\alpha } } \right] Var\left[ \alpha \right] } }}$$

where $\alpha$ represents the true value and $\hat{\alpha }$ represents the predicted value.

### Performance evaluation on simulated dataset

To demonstrate and evaluate DCap, we first carried out simulation experiments. Two single-cell datasets E-MTAB-5061 [16] and GSE81608 [17] were used in the simulation experiment.

#### Simulation dataset generation

The method has two inputs: a bulk RNA-Seq dataset and a single-cell RNA-seq dataset. The single-cell RNA-seq dataset is E-MTAB-5061. We use another single-cell RNA-seq dataset, the GSE81608 dataset, to generate the bulk RNA-Seq dataset.

The GSE81608 dataset contains 18 samples (12 normal samples and 6 T2D diseases samples). If every sample is a bulk RNA-Seq data, we can obtain a dataset containing 18 bulk RNA-Seq data. The gene expression matrix of all cells from the same sample is merged to obtain the gene expression matrix of the bulk RNA-Seq data. Then, we record the number of cells of each type in each bulk RNA-Seq data to provide ground truth for the subsequent evaluation method.

#### Experimental results

To perform benchmark tests systematically, we first applied DCap and four other methods (Nonnegative least squares (NNLS), MuSiC, CIBERSORT, and BSEQ-sc) to the simulated dataset to obtain the predicted cell abundance. We use three metrics (RMSD, mAD, R) to evaluate the results of different methods. Table 2 shows that DCap performs the best among the five methods on all three evaluation metrics. The RMSD and mAD values of DCap are the smallest, and the R-value of DCap is the highest among the five methods.

**Table 2:** Error analysis of prediction results

Method	RMSD	mAD	R
DCap	0.08	0.05	0.96
NNLS	0.11	0.08	0.90
MuSiC	0.10	0.06	0.93
BSEQ-sc	0.21	0.15	0.79
CIBERSORTS	0.21	0.15	0.76

**Fig. 1:**
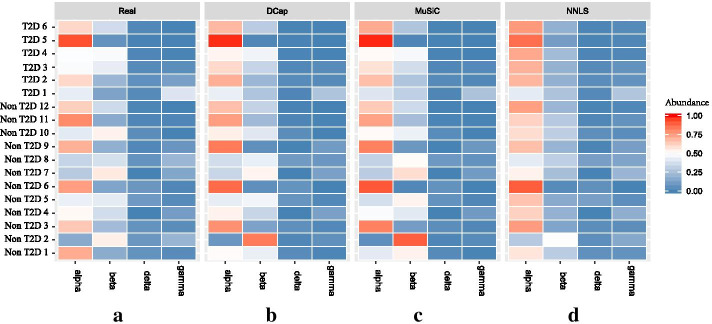
Heat map of the real value and the estimated value obtained by different methods. A heat map of the real values and the estimated values obtained by different methods. The horizontal axis represents the cell type and the vertical axis represents the name of the simulated bulk tissue. The shade of color indicates the proportion of a cell type in bulk tissue. By the heat map, we can observe the comparison of the predicted results with the actual values for each bulk tissue and cell type. (a)real value. (b)The results of DCap prediction. (c)The results of MuSiC prediction (d)The results of NNLS prediction

**Fig. 2:**
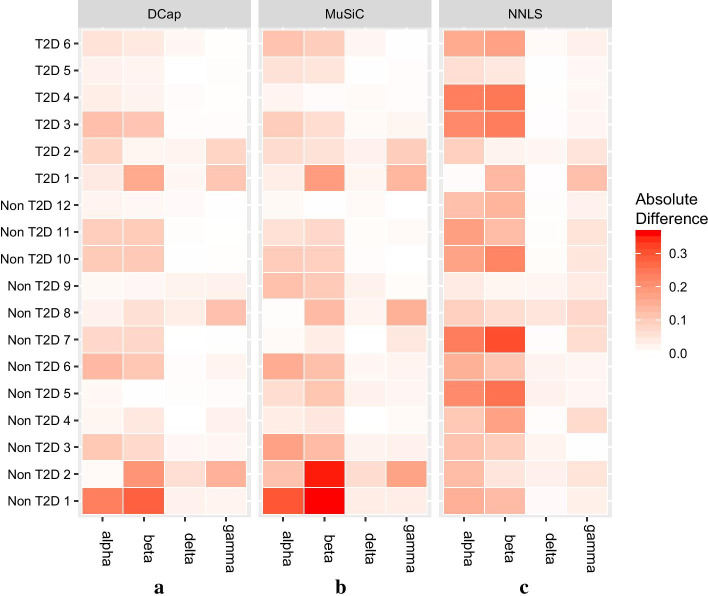
Heat map of the absolute difference between predicted value and true value. A heat map of the absolute difference between the predicted value and the true values. The horizontal axis represents the cell type, and the vertical axis represents the name of the simulated bulk tissue. The shade of color indicates the absolute difference between the predicted value and the real value for the proportion of a cell type in bulk tissue. By the heat map, we can observe the predicted results of each bulk tissue and cell type. The lighter the color, the closer it is to the true value. **a** DCap, **b** MuSiC, **c** NNLS

To compare with ground truth data, we visualize ground truth data and the prediction results of the three algorithms (DCap, MuSiC, and NNLS) in Fig. 1. The result show that DCAP performs the best among three methods. We made the heat map of the absolute difference between the predicted value and ground truth in Fig. 2.

**Fig. 3:**
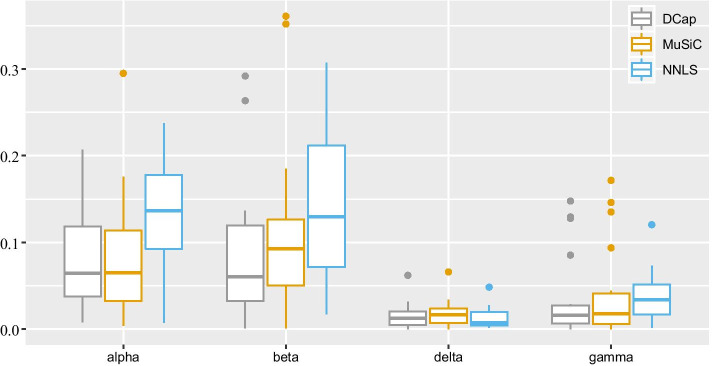
Boxplot of the absolute difference between predicted value and true value. A boxplot of the absolute difference between the predicted value and the true value. The horizontal axis represents the cell type, and the vertical axis represents the absolute difference between the predicted value and the true value. Each color represents a method

**Fig. 4:**
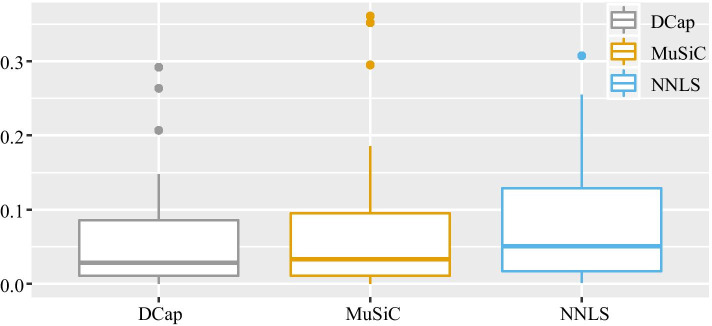
Boxplot of the total absolute difference between predicted value and true value. A boxplot of the total absolute difference between the predicted value and the true value. The horizontal axis represents the method type, and the vertical axis represents the absolute difference between the predicted true and the real value. Each color represents a method

Figure 2 shows that DCap is superior to the other two methods. To understand the comparison between DCap and other methods more clearly, we made the boxplot of the difference between the predicted value and ground truth of each cell type, shown in Fig. 3. A smaller difference between the predicted value and true value represents better results. Finally, we aggregate the absolute difference of the same method and made the boxplot of the absolute difference of each method in Fig. 4. Figure 4 shows that the total absolute difference between the predicted value and the true value of DCap is the smallest. DCap performs better than other methods in general.

### Cell proportion prediction on real dataset

We applied the model to real bulk RNA-seq dataset to analyze the proportion of various types of cells in real tissues.

We used GSE50244 [18], which is the bulk RNA-Seq dataset, and E-MTAB-5061, which is the single-cell RNA dataset, as input. The GSE50244 dataset contains gene expression data of 89 islet samples.

**Fig. 5:**
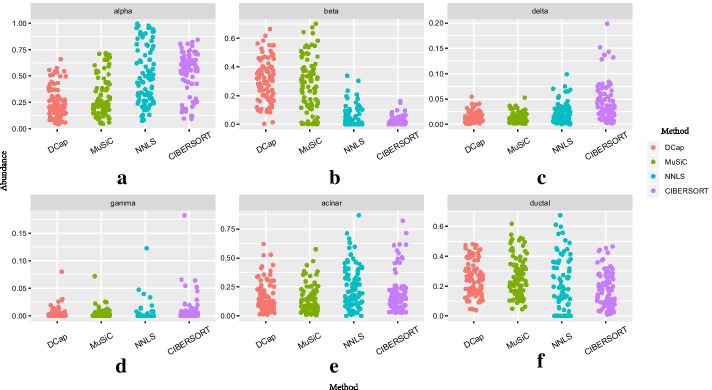
Dithering plot of predicted cell abundance. The predicted dithering plot of cell proportion. Each one corresponds to a cell type. The horizontal axis of the small graph represents the method type, and the vertical axis represents cell proportion. Each dot represents the percentage of cells predicted by a particular type of cell in this islet tissue. Each small diagram contains 89 dots, and every dot represents an islet tissue. Each color represents a method. **a** Alpha cell prediction results, **b** beta cell prediction results, **c** delta cell prediction results, **d** gamma cell prediction results, **e** acinar cell prediction results, **f** ductal cell prediction results

By applying DCap and three other methods, we estimate the proportion of the 6 main cell types in the islet: alpha, beta, delta, gamma, acinar and ductal, which account for more than $90$ $\%$ of the whole islet’s cells. The relative abundance of cell types is shown in Fig. 5.

The results show that the proportion of beta cells is the largest, which is also in line with the the known biomedical knowledge. The results of all the four methods show that the proportion of gamma cells is the least.

**Fig. 6:**
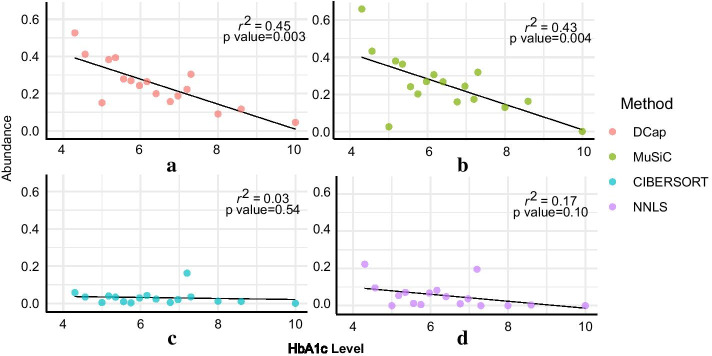
Ratio of HbA1c to beta cells. The ratio of HbA1c levels to predicted beta cells. Each little diagram corresponds to a method. The horizontal axis of the small figure is the HbA1c level, and the vertical axis is the proportion of beta cells in each islet tissue. **a** DCap, **b** MuSiC, **c** NNLS, **d** CIBERSORT

## Discussion

The prevalence of type 2 diabetes mellitus (T2D) is generally determined by the level of HbA1c. When the patient’s HbA1c level was greater than 6.5$\%$, the patient was diagnosed with T2D. With the progression of T2D, the number of beta cells decreases gradually. As the HbA1c level increases, the number of beta cells decreased gradually.

We evaluated the performance of DCap from the cell changes caused by T2D disease. Based on the proportion of beta cells in all islet tissues and the corresponding HbA1c level, a regression curve can be obtained by linear regression. The linear regression method can be measured by $r^2$ and p values. In detail, $r^2$ ranges from 0 to 1. The closer $r^2$ gets to 1, the better performance it represents. The smaller p-value represents the more reliability of the linear regression model. Therefore, we performed regression modeling in Fig. 6.

Figure 6 indicates that the proportion of beta cells predicted by DCap is correlated with the HbA1C level. DCap has a better $r^2$ and smaller P-value, which shows that DCap’s prediction results are generally better than the other three methods.

**Fig. 7:**
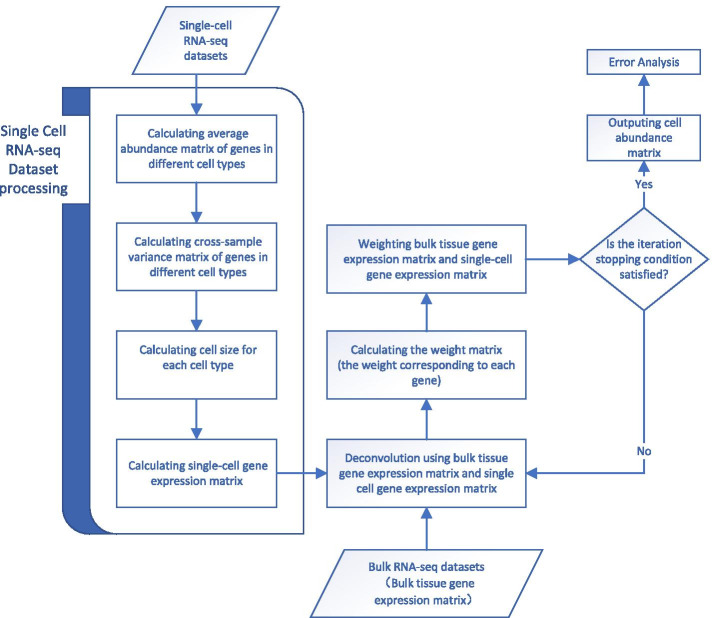
DCap workflow. The workflow of DCap. There are two conditions for stopping the iteration. If any one of the conditions is met, the iteration stops. (1) The difference between the predicted cell abundance matrix and the previously predicted cell abundance matrix is less than the given threshold. (2) The number of iterations is equal to the given threshold

**Fig. 8:**
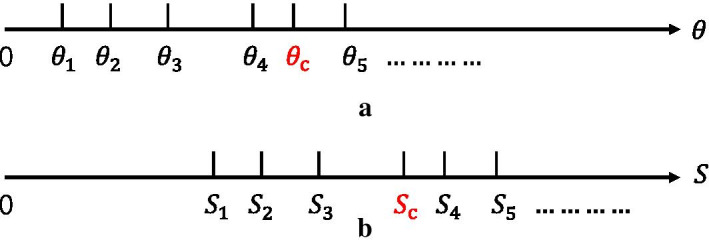
Clustering diagram. **a** $\theta$ clustering example diagram. **b** S clustering example diagram

## Conclusion

We proposed a novel method, named Dcap, to predict cell abundance. Compared with most other methods, DCap does not need a single-cell reference matrix in advance. It reduces the difficulty of cell abundance prediction. It only needs bulk RNA-seq datasets of tissue gene expression and corresponding single-cell RNA-seq datasets to predict cell abundance. The result shows that DCap performs better than other methods. We can study the changes of cell abundance in diseased tissues better and provide more information for the following treatment of diseases. Inspired by the success of deep learning methods in biomedical data analysis [19–22], we will apply deep learning methods to predict cell abundance in the future.

## Method

The flow chart of DCap is shown in Fig. 7.

The inputs of DCap are bulk RNA-seq datasets and single-cell RNA-seq datasets. First, the single-cell dataset is used to obtain the single-cell gene expression matrix and the cross-cellular variance matrix of the gene for the deconvolution. Then, the bulk tissue gene expression matrix and the single-cell gene expression matrix are deconvolved. The weighted matrix is calculated by these two matrices. Finally, the weighted matrix is used for deconvolution, and the aforementioned steps are repeated until the result converges.

### Single-cell RNA-seq dataset processing

The single-cell RNA-seq technology can measure gene expression profiles at the cell level. A single-cell RNA-seq dataset often contains cells of multiple types from multiple samples (subjects). For example, mouse kidney cell data from Park et al. [23] was derived from seven healthy mouse kidneys containing 16 types of 43,745 cells. Each cell contains the expression value of 16,273 genes. Therefore, it is necessary to select cell types according to the input data to be deconvoluted. Then we generated a single-cell gene expression matrix based on single-cell RNA-seq datasets. The generated matrix includes the expression profile of each gene at different types of cell types. Each row in the matrix represents a gene. Each column in the matrix represents a cell type. Therefore, the quality of the single-cell RNA-seq dataset process is important for predicting cell abundance.

#### Calculating average abundance matrix of genes

Each row of the average abundance matrix represents a gene. Each column represents a cell type. The value in the matrix represents the average abundance of a certain gene in a certain type of cell.

In tissue *j*, the relative abundance of gene *g* in cells of type *k* is $\theta _{jg}^K$. $Y_{jgc}$ is the number of mRNA molecules of gene *g* in cell *c*. $C_j^k$ is the set of cell index for cell type *k*. $\theta _{jg}^k$ is calculated as:

4 $$\begin{aligned} \theta _{jg}^k = \frac{{\mathop \sum \nolimits _{c \in C_j^k} {Y_{jgc}}}}{{\mathop \sum \nolimits _{c \in C_j^k} \mathop \sum \nolimits _{g'=1}^G {Y_{jg'c}}}} \end{aligned}$$

The single-cell RNA-seq dataset contains multiple tissues from different subjects, and $\theta _{jg}^k$ is different for different subjects. Therefore, we first calculate $\theta _{jg}^k$ for tissue cells of each subject. The final gene relative abundance $\theta _{g}^{k'}$ is the average of $\theta _{jg}^k$ across different subjects. Considering the existence of abnormal values, we firstly determine the abnormal values before calculating the final gene relative abundance.

As shown in Fig. 8a, all values of $\theta$ are placed on a number axis. The K-means clustering method is used to group all the values into different clusters to find the center point $\theta _c$. Then, the outliers are removed based on the distance from the center point. Let the set distance threshold be $\rho _{\theta }$, then

5 $$\begin{aligned} \theta _{jg}^{k'} = \frac{{\mathop \sum \nolimits _{j = 1}^J \theta _{jg}^k}}{J_\theta } \end{aligned}$$

where, $\left| {\theta _{jg}^k - \theta _c} \right| < \rho _{\theta }$, $J_\theta$ is the number of $\theta$ after excluding outliers. Generally, $\rho _{\theta }$ is selected as the most suitable value by means of grid searching technique.

#### Calculating cross-sample variance matrix of genes in different cell types

Rows of the cross-sample variance matrix of genes represent genes. Columns represent different cell types. The values in the matrix represent the variance of the expression of a gene in different samples in a certain cell type.

In tissue *j*, the variance of gene *g* expression in different samples in cells of type *k* is $V_{jg}^K$. $V_{jg}^k$ is calculated as:

6 $$\begin{aligned} V_{jg}^k = Var\left[ {\theta _{jg}^k} \right] \end{aligned}$$

#### Calculating cell size for each cell type

The value in the cell size vector of each tissue represents the average number of RNA molecules for each cell type.

For tissue *j*, let $m_{j}^k=|C_{j}^k|$ be the total number of cells of type *k* and $S_{j}^k$ be the average of the total number of RNA molecules for cells of type *k*. $S_j^k$ is calculated as:

7 $$\begin{aligned} S_j^k = \frac{{\mathop \sum \nolimits _{c \in C_j^k} \mathop \sum \nolimits _{g' = 1}^G {Y_{jg'c}}}}{{m_j^k}} \end{aligned}$$

For different subjects, $S_{j}^k$ are different. Therefore, we first calculate $S_{j}^k$ for each subject. The final gene relative abundance $S_{j}^{K'}$ is the average $S_{j}^k$ across different subjects. As shown in Fig. 8b, all values of *S* are placed on a number axis. The K-means clustering method is used to group all the values into different clusters to find the center point $S_c$. Outliers are removed by the method introduced in the previous subsection.

Let the set distance threshold be $\rho _{s}$, then

8 $$\begin{aligned} S _{j}^{k'} = \frac{{\mathop \sum \nolimits _{j = 1}^J S _{jg}^k}}{J_S} \end{aligned}$$

where, $\left| {S _{jg}^k - S _c} \right| < \rho _{s}$, $J_S$ is the number of *S* without outliers. Generally, $\rho _{s}$ is selected as the most suitable value by means of grid searching technique.

#### Calculating single-cell gene expression matrix

Rows of the single-cell gene expression matrix represent different genes. Columns represent different cell types. The values in the matrix represent the expression level of genes in a certain type of cell.

Let $Y_{jg}$ be the total number of mRNA molecules of gene *g* in a given tissue *j*, consisting of *K* types of cells. $Y_{jg}$ are calculated as:

9 $$\begin{aligned} {Y_{jg}} = \mathop \sum \limits _{k = 1}^K \mathop \sum \limits _{c \in C_j^k} {Y_{jgc}} \end{aligned}$$

Based on Eqs. (1)–(6), $Y_{jg}$ can be represented as:

10 $$\begin{aligned} {Y_{jg}} = \mathop \sum \limits _{k = 1}^K m_j^kS_j^{k'}\theta _{jg}^{k'} \end{aligned}$$

Let ${m_j} = \mathop \sum \limits _{k = 1}^K m_j^k$ be the total number of cells in tissue *j*. Let $p_j^k = \frac{{m_j^k}}{{{m_j}}}$ be the proportion of cells of type *k* in tissue *j*. $\frac{{{Y_{jg}}}}{{{m_j}}}$ is calculated as:

11 $$\begin{aligned} \frac{{{Y_{jg}}}}{{{m_j}}} = \mathop \sum \limits _{k = 1}^K p_j^kS_j^{k'}\theta _{jg}^{k'} \end{aligned}$$

The gene expression level of the gene *g* in the cells of type *k* is $X_g^k$. $X_g^k$ is calculated as:

12 $$\begin{aligned} X_{jg}^k = S^{k'}\theta _{jg}^{k'} \end{aligned}$$

### Weighted matrix equation derivation

Considering Eq. (6), in the absence of error, we can directly use $Y_g^k$ and $X_g^k$ to find $p_j^k$. However, in actual cases, when we use the bulk RNA-seq to obtain $Y_g^k$, there is measurement noise. Therefore, we need to modify Eq. (6). In order to guarantee the condition of $\mathop \sum \nolimits _{k = 1}^K p_j^k=1$, the adjustment parameter *C* is added to the equation.

13 $$\begin{aligned} {Y_{jg}} = {C_j}(\mathop \sum \limits _{k = 1}^K p_j^kX_{jg}^k + {\epsilon _{jg}}) \end{aligned}$$

where, $\epsilon _{j g} \sim N\left( 0, \delta _{j g}^{2}\right)$ represents the measurement error of bulk RNA-seq.

After $X_{jg}$ and $p_j$ are calculated, the variance between the actual value of $Y_{jg}$ and the estimated value is:

14 $$\begin{aligned} Var\left[ {{Y_{jg}}|{p_j},{X_{jg}}} \right] = {C_j}^2\delta _{jg}^2 \end{aligned}$$

In addition to the measurement error that occurs during the bulk RNA-seq process, there is also an error in generating the single-cell reference matrix $X_g^k$. In different samples (eg, unified tissues derived from different subjects), the same type of cells have different gene expression levels.

We define a gene with a small variance of expression in the same cell type between different samples as an information gene. The expression of the information gene is stable in this cell type. Genes with a large variance of expression in the same cell type between different samples are defined as non-information genes. Therefore, the relative abundance of gene *g* in cells of type *k* may not be a unique value in the calculation of a single-cell reference matrix across different samples.

Both types of errors are important. Both types of errors may happen during the process of obtaining data. The importance of different types of errors may be different for different datasets. In DCap, the weight of these two types of errors is considered the same. We use the sum of these two types of errors as weight information to improve prediction accuracy. So we can calculate the variance of the actual value of $Y_{jg}$ and the estimated value $p_j$ is:

15 $$\begin{aligned} Var\left[ {{Y_{jg}}|{p_j}} \right]& {} = {C_j}^2\delta _{jg}^2 + Var\left[ {{C_j} \cdot \mathop \sum \limits _{k = 1}^K p_j^kX_{jg}^k} \right] \nonumber \\& {}= {C_j}^2\delta _{jg}^2 + {C_j}^2 \cdot \mathop \sum \limits _{k = 1}^K p_{jk}^2S_j^{k'2}Var\left[ {\theta _{jg}^k} \right] \nonumber \\& {}= {C_j}^2\delta _{jg}^2 + {C_j}^2 \cdot \mathop \sum \limits _{k = 1}^K p_{jk}^2S_j^{k'2}v_{gk}^2 \end{aligned}$$

where $V_{gk}$ is the variance of the expression of gene *g* in different samples for type *k* cells.

Therefore, for the tissue *j*, $w_{jg}$ is calculated as:

16 $$\begin{aligned} \frac{1}{{{w_{jg}}}} = Var\left[ {{Y_{jg}}|{p_j}} \right] = {C_j}^2\delta _{jg}^2 + {C_j}^2 \cdot \mathop \sum \limits _{k = 1}^K p_{jk}^2S_k^{'2}v_{gk}^2 \end{aligned}$$

Considering the case of $Var\left[ {{Y_{jg}}|{p_j}} \right] =0$, the adjustment parameter *n* is added to the equation 11 to calculate the final weight:

17 $$\begin{aligned} \frac{1}{{{w_{jg}}}} =n+{C_j}^2\delta _{jg}^2 + {C_j}^2 \cdot \mathop \sum \limits _{k = 1}^K p_{jk}^2S_k^{'2}v_{gk}^2 \end{aligned}$$

Weighting the two matrices during the deconvolution process can reduce errors and improve the accuracy of the estimates. However, in the actual case, $\delta _{jg}^2$ is unknown. Therefore, we start from non-negative least squares and use iteration to estimate the weight until convergence.

### Deconvolution equation derivation

Based on Eqs. (6) and (7), $Y_{jg}$ is calculated as:

18 $$\begin{aligned} {Y_{jg}} = {m_j}\mathop \sum \limits _{k = 1}^K p_j^kX_g^k \end{aligned}$$

Then we multiplied the weights to both sides of Eq. (15):

19 $$\begin{aligned} \sqrt{{w_{jg}}} {Y_{jg}} = \sqrt{{w_{jg}}} {m_j}\mathop \sum \limits _{k = 1}^K p_j^kX_g^k \end{aligned}$$

Let *A*, *B*, and *C* be three matrices, where $A = \frac{{\sqrt{{w_{jg}}} {Y_j}}}{{{m_j}}}$, $B=p_j$, $C = \sqrt{{w_{jg}}} X$. The problem can be defined as calculating the *B* matrix when $mi{n_A}\left( {BC - {A^2}} \right)$, which is also the problem of least squares solution.

After inputting the single-cell dataset, we use Eq. (10) to calculate the single-cell reference matrix.

The gene expression matrix *Y* usually contains gene expression of multiple tissues. We predict each tissue separately and integrate the results into one matrix.

## Data Availability

Data analyzed in this study were a re-analysis of existing data, which are openly available at locations cited in the reference section. E-MTAB-5061 dataset has been deposited in ArrayExpress (EBI) with links: https://www.ebi.ac.uk/arrayexpress/experiments/E-MTAB-5061/ GSE50244 dataset has been deposited in the NCBI GEO with links: https://www.ncbi.nlm.nih.gov//geo/query/acc.cgi?acc=GSE50244 GSE81608 dataset has been deposited in the NCBI GEO with links: https://www.ncbi.nlm.nih.gov//geo/query/acc.cgi?acc=GSE81608.

## Abbreviations

SVR: Support vector regression
SVM: Support vector machine
NNLS: Nonnegative least squares
RMSD: Root-mean-square Deviation
mAD: Mean absolute difference
T2D: Type 2 diabetes
